# Telemedicine for the management of diabetic patients in a high-complexity Latin American hospital

**DOI:** 10.1186/s12913-023-09267-0

**Published:** 2023-03-30

**Authors:** Luz Angela Casas, Juliana Alarcón, Alejandra Urbano, Evelyn E. Peña-Zárate, Saveria Sangiovanni, Laura Libreros-Peña, María Fernanda Escobar

**Affiliations:** 1 Departamento de Endocrinología, Fundación Valle del Lili, Cali, Colombia; 2 Centro de Investigaciones Clínicas, Fundación Valle del Lili, Cali, Colombia; 3 Departamento de Telemedicina, Fundación Valle del Lili, Cra 98 Nro. 18-49, Cali, 760032 Colombia; 4 Facultad de Ciencias de la Salud, Universidad Icesi, Cali, Colombia

**Keywords:** Telemedicine, Telehealth, Diabetes, Metabolic control, Glycosylated hemoglobin

## Abstract

**Background:**

Noncommunicable diseases such as diabetes mellitus (DM) have gained attention worldwide. Latin America experienced a rise in rates of DM. During the COVID-19 pandemic, a telemedicine program was implemented in a quaternary care academic complex in Latin America to continue the follow-up of patients with diabetes.

**Objective:**

The aim of this study is to describe the clinical experience of DM patient management through telemedicine and the HbA1c behavior of patients followed-up through this modality.

**Materials and methods:**

We conducted a retrospective cohort study including all patients with type 1 or 2 diabetes who were treated via telemedicine from March to December 2020. A Wilcoxon statistical test was used to compare the changes in glycosylated hemoglobin between the first teleconsultation and after 6 months of telemedicine follow-up.

**Results:**

A total of 663 patients were included, 17.65% (117) of whom had type 1 diabetes and 82.35% (546) of whom had type 2 diabetes. Patients with both types of diabetes, presented with stable HbA1c values regardless of the length of follow-up.

**Conclusion:**

The use of telemedicine can be a helpful tool for both patients and health care providers to support the continuity of care to maintain acceptable control levels within glycemic control goals.

## Introduction

Diabetes affects approximately 1,676,885 patients in Colombia, with the highest prevalence found in the country’s central region [1]. Despite type 2 diabetes being the most prevalent, type 1 diabetes arises mostly in children and accounts for 5–10% of all diabetes types. Diabetes is associated with various conditions, such as cardiovascular disease, renal failure, lower limb amputations, retinopathy, and neuropathy [2, 3]. To avoid complications related to diabetes, guidelines suggest lifestyle habits, maintaining optimal blood glucose levels according to individual glucose targets, and medication adherence [4, 5].

For several decades, the development of communication strategies and tools has been proposed to provide health care and support the management of chronic diseases such as diabetes mellitus (DM) [6]. In 2020, as the COVID- 19 pandemic rapidly increased worldwide and due to the lockdown and preventive measures implemented in Colombia to avoid its spread [7], there was a reduction in in-person outpatient care, and telemedicine emerged as an alternative to continue the provision of outpatient care services [8, 9].

Telemedicine or telehealth uses telecommunications to support different areas of health care, becoming a fundamental tool to reduce barriers to health access, with a high level of satisfaction for patients and health personnel [10]. Despite the limitation of telemedicine for physical examination, it can be a useful tool for the management and follow-up of patients with diabetes; it can even improve the glycemic control of these patients [11–13]. In the present study, we describe the changes in glycosylated hemoglobin (HbA1c) in patients with type 1 and type 2 diabetes patients seen by teleconsultation.

## Materials and methods

### Design

We conducted a retrospective cohort study that included patients of all ages with a diagnosis of type 1 and type 2 diabetes mellitus who were followed through endocrinology teleconsultation from March 1 to December 31, 2020, at the Fundación Valle del Lili (FVL). Patients were excluded if they required in-person medical care due to their clinical condition or if the taking of vital signs and the physical examination were essential for decision-making.

### Overview of the “Siempre” teleconsultation program

The “Siempre” program, which in English means “always” was designed as an alternative for outpatient care during the COVID-19 pandemic given the lockdown. This program allows communication between the endocrinologist and the patient by synchronous video call using the Microsoft Teams® platform. During the teleconsultation, the patient’s current status is inquired, laboratory and imaging tests are reviewed, adherence to current medication is inquired, and ambulatory blood glucose monitoring records are requested. All teleconsultation information was recorded in the institutional clinical record system (SAP). Subsequently, a summary of the appointment, clinical orders, and medical prescriptions in PDF format are sent to the patient’s email.

Before ending the teleconsultation, all patients were informed about lifestyle habits (healthy diet, regular physical activity, etc.), diabetes foot care, the importance of adherence to pharmacological management, and being informed about warning signs. Patients with a glucometer at home were instructed on its use for self-monitoring of glucose levels. The HbA1c target, treatment, and follow-up for each patient were determined according to the American Diabetes Association guidelines [14–19].

### Variables

The data were collected retrospectively from the institutional medical records and registered in a database created in the BDClinic platform. Sociodemographic and clinical data collected included sex, age, type of health insurance, and comorbidities. The change in HbA1c was assessed using the values at the first and last teleconsultation performed during the study period.

### Statistical analysis

The information was collected by trained personnel while other unrelated personnel randomly performed a quality audit by comparing the information recorded with the clinical history. An exploratory analysis was performed to detect missing data and extreme values, followed by a descriptive analysis of the data; quantitative variables were expressed as the median and interquartile range (IQR) due to nonnormality. To determine the change in glycosylated hemoglobin and the other metabolic parameters (cholesterol, LDL cholesterol, HDL cholesterol, triglycerides, creatinine, and microalbuminuria) between the first and last teleconsultation the follow-up time was stratified (< 6 months and ≥ 6 months) and a Wilcoxon test was used due to the nonnormality of the data. The results were categorized by type of diabetes and were stratified according to follow-up time. Results with p values less than 0.05 were considered statistically significant. All analyses were performed using Stata 17.

## Results

**Fig. 1:**
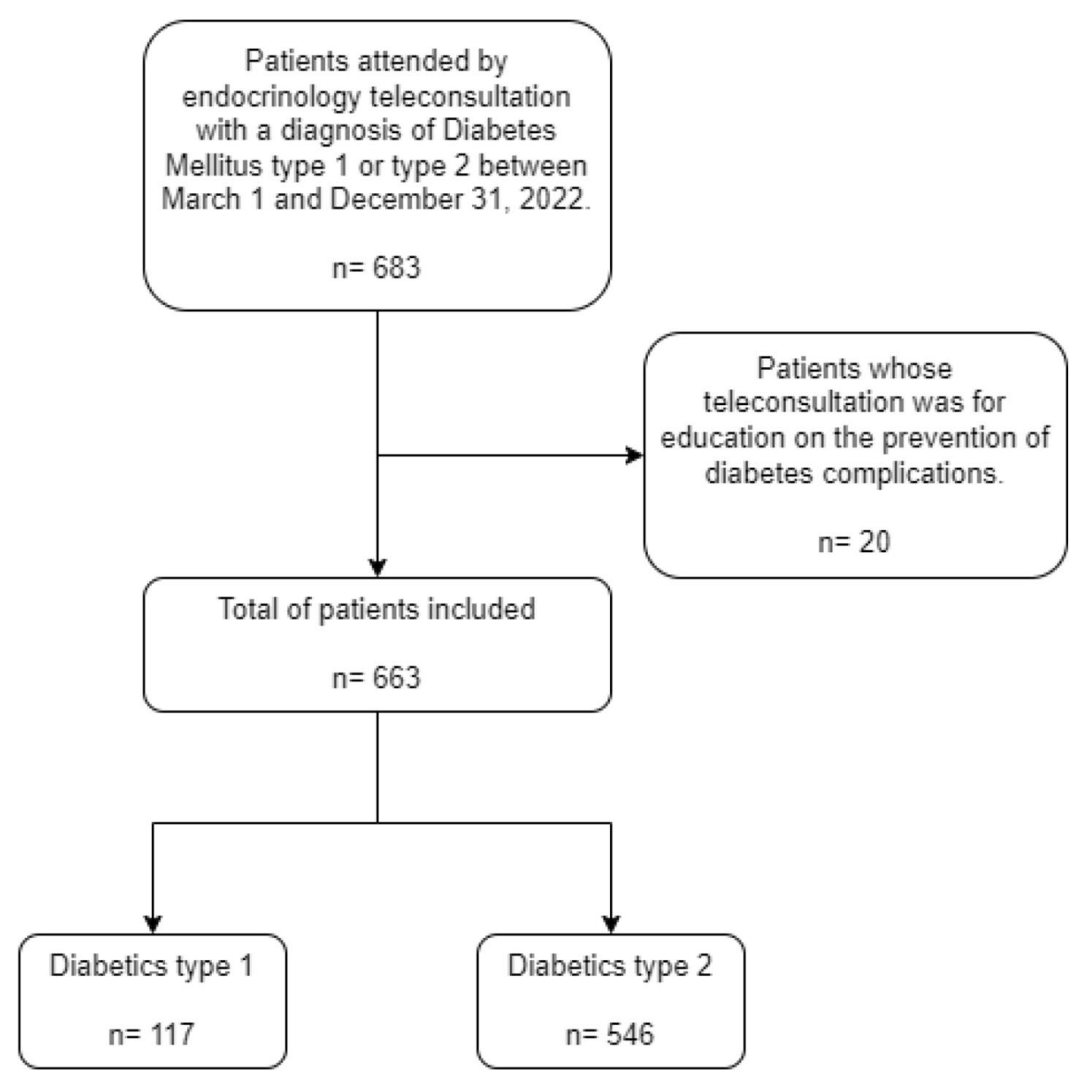
Flowchart of included patients

Of the 683 patients with type 1 and type 2 diabetes who attended the endocrinology teleconsultation during the study period, 20 were excluded because the medical history of the teleconsultation, indicated that they were patients with prediabetes who received diabetes prevention education; thus, a total of 663 patients were obtained for the analysis, 117 (17.65%) with type 1 diabetes and 546 (82.35%) with type 2 diabetes (Fig. 1).

The median age was lower in patients with type 1 diabetes (20 [15–32] vs. 67 [58–75]), and the time in years since the diagnosis of diabetes was 8 (3–15) years for those with type 1 diabetes and 9 (3–15) years for those with type 2 diabetes. In both groups, less than half of the patients had microvascular or macrovascular complications related to diabetes (18/117, 15.38% and 174/546, 31.86%), the most frequent being chronic kidney disease. More than 90% of patients with type 1 diabetes were treated with insulin, and in patients with type 2 diabetes the most commonly used drugs was metformin (379, 69.41%), followed by DPP-4 (204, 37.36%) and SGLT2 (198, 36.2%) (Table 1).

**Table 1:** Demographic and clinical characteristics

Variable	Diabetes Mellitus	
	Type 1, n = 117	Type 2, n = 546
Age, years*	20 (15–32)	67 (58–75)
Time since diabetes diagnosis, years*	8 (3–15)	9 (3–15)
Area of residence (%)		
Urban	81 (69.2)	443 (81.1)
Rural	4 (3.4)	17 (3.1)
History of hypertension (%)	8 (6.8)	315 (57.6)
Microvascular and macrovascular complications (%)		
No	94 (80.34)	348 (63.73)
Yes	18 (15.38)	174 (31.86)
Chronic kidney disease	13 (11.11)	76 (13.91)
Neuropathy	11 (9.40)	37 (6.77)
Retinopathy	9 (7.69)	42 (7.69)
Peripheral vascular disease	1 (0.85)	16 (2.93)
Coronary artery disease	0	72 (13.18)
Antidiabetic drugs (%)		
No	0	33 (6.04)
Yes	117 (100)	512 (93.77)
Metformin	8 (6.83)	379 (69.41)
Sulfonylureas	0	12 (2.19)
Dipeptidyl peptidase 4 (DPP-4) inhibitors	4 (3.41)	204 (37.36)
Insulin	110 (94.01)	176 (32.23)
GLP-1 analog	7 (6)	67 (12.27)
Sodium-glucose cotransporter (SGLT2) inhibitors	13 (11.1)	198 (36.2)

* Median, interquartile range

Table 2 shows that 99 (18.1%) of the patients with type 2 diabetes had their last teleconsultation in the first 6 months after the first teleconsultation, and 217 (39.7%) had their last teleconsultation 6 months or more after the first teleconsultation. The HbA1c levels remained stable between the first and last teleconsultation in patients with type 2 diabetes (6.6 [6.1–7.3] vs. 6.6 [6.1–7.3]). Of the patients with type 1 diabetes 52 (44.4%) had their last teleconsultation follow-up at 6 months or later. The HbA1c levels decreased from a median of 7.7 (7-8.7) at the first teleconsultation to 7.5 (7-8.67) at the last teleconsultation in the group of patients with type 1 diabetes.

**Table 2:** Values at the first and last teleconsultation by type of diabetes

Variable	Diabetes Mellitus	
	Type 1, n = 117	Type 2, n = 546
Time between the first and last teleconsultation		
< 6 months	19 (16,2)	99 (18,1)
≥ 6 months	52 (44,4)	217 (39,7)
ND (Patients with only one teleconsultation evaluation)	46 (39,3)	230 (42,1)
Glycosylated hemoglobin, %*		
First teleconsultation	7.7 (7-8.7)	6.6 (6.1–7.3)
Last teleconsultation	7.5 (7-8.67)	6.6 (6.1–7.3)
LDL cholesterol, mg/dL*		
First teleconsultation	99 (82–122)	82 (59–116)
Last teleconsultation	102 (75–123)	77 (57–103)
HDL cholesterol, mg/dL*		
First teleconsultation	62 (49–73)	45 (37–53)
Last teleconsultation	56 (50–70)	45 (38–53)
Triglycerides, mg/dL*		
First teleconsultation	86.5 (57–112)	140 (104–189)
Last teleconsultation	88 (62–100)	133 (96–163)
Creatinine, mg/dL*		
First teleconsultation	0.745 (0.6–0.92)	0.865 (0.7–1.06)
Last teleconsultation	0.8 (0.73–0.93)	0.89 (0.76–1.08)
Microalbuminuria, mg/L*		
First teleconsultation	5.2 (3-16.5)	7.5 (3.6–28)
Last teleconsultation	10.2 (4.4–19.5)	10 (4.4–31)

* Median, interquartile range. ND: no data

**Fig. 2:**
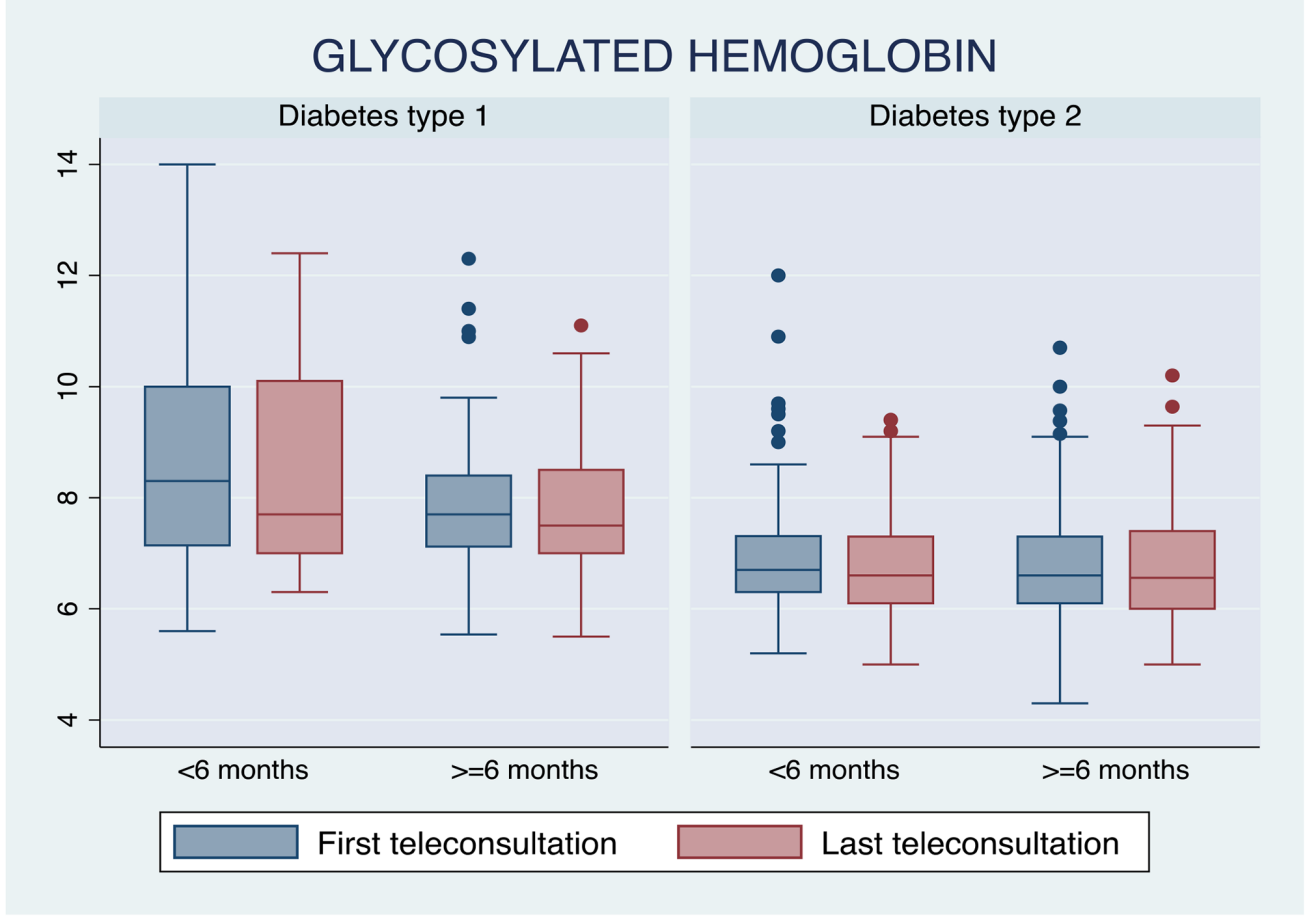
Changes in HbA1c between the first and last teleconsultation by type of diabetes and follow-up time

**Fig. 3:**
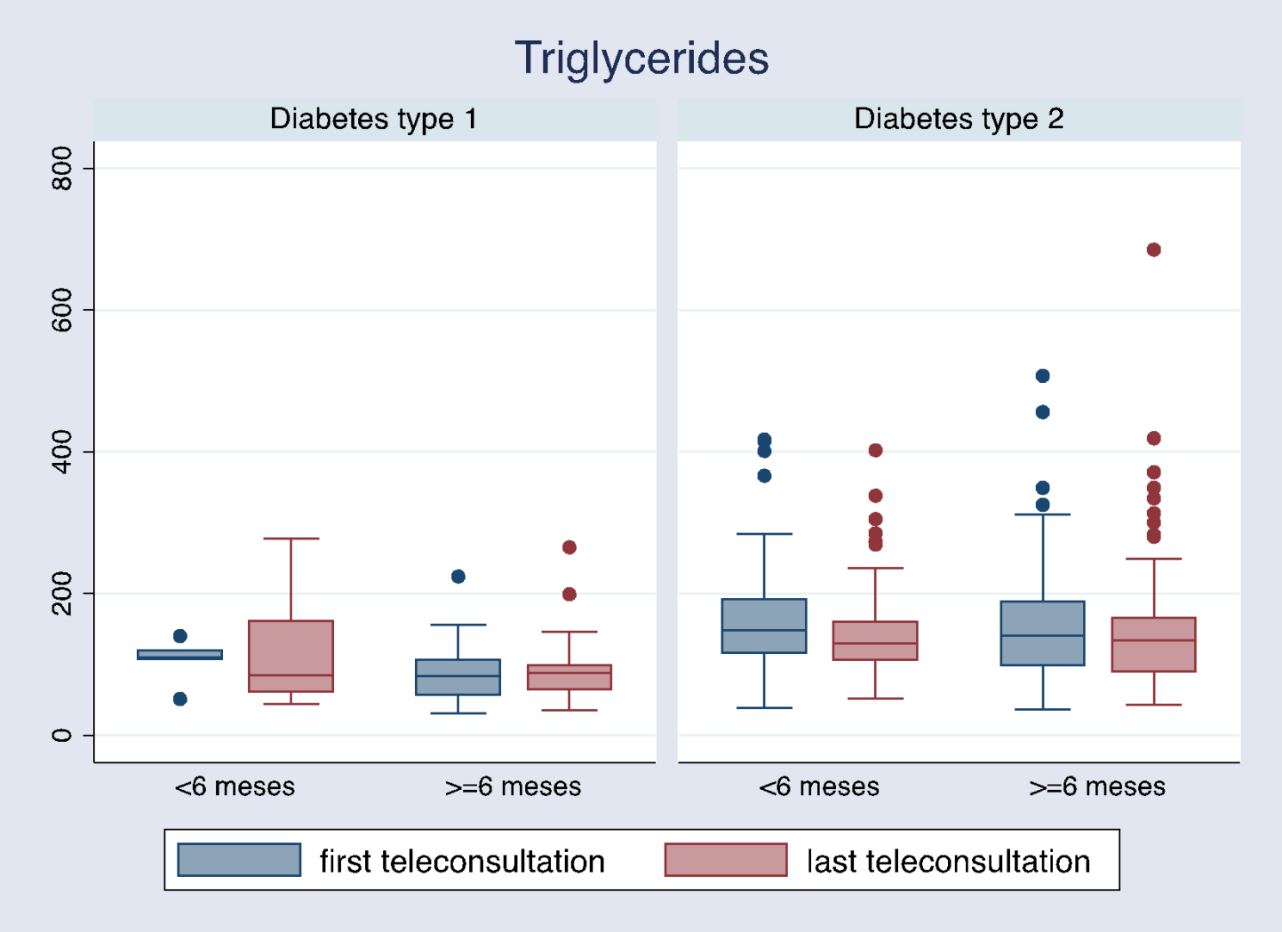
Changes in triglycerides levels between the first and last teleconsultation by type of diabetes and follow-up time

Patients with type 2 diabetes and a follow-up length of fewer than 6 months had no significant changes in glycosylated hemoglobin (6.6 [6.1–7.3] vs. 6.5 [6-7.4], p = 0.858) as well as patients with a follow-up equal to or greater than 6 months (6.6 [6.1–7.3] vs. 6.5 [6-7.4], p = 0.858) (Table 3; Fig. 2). Additionally, changes in other laboratory parameters between the first and the last teleconsultation can be observed (Table 3), with a significant reduction in triglycerides in patients where follow-up was equal to or greater than 6 months (142.5 [106-191.5] vs. 135.5 [90.5–166], p = < 0.001) (Fig. 3).

**Table 3:** Differences between the first and last teleconsultation metabolic parameter values, stratified by follow-up time in patients with type 2 diabetes

Type 2 Diabetes	First Teleconsultation	Last teleconsultation	p value	n
Glycosylated hemoglobin, %*				
< 6 months	6.7 (6.3–7.2)	6.6 (6.1–7.3)	0.353	67
≥ 6 months	6.6 (6.1–7.3)	6.5 (6-7.4)	0.858	189
LDL cholesterol, mg/dL*				
< 6 months	87 (61–111)	81 (59–101)	0.242	45
≥ 6 months	80.5 (59–112)	76.8 (57.5–100)	0.053	148
HDL cholesterol, mg/dL*				
< 6 months	42.5 (38–50)	42.5 (38–50)	0.688	42
≥ 6 months	46 (38–53)	46 (37–53)	0.135	133
Triglycerides, mg/dL*				
< 6 months	141 (117–211)	134 (106–173)	0.317	47
≥ 6 months	142.5 (106-191.5)	135.5 (90.5–166)	< 0.001	144
Creatinine, mg/dL*				
< 6 months	0.95 (0.715–1.16)	0.915 (0.74–1.24)	0.981	44
≥ 6 months	0.84 (0.71–1.07)	0.89 (0.76–1.08)	< 0.001	131
Microalbuminuria, mg/L*				
< 6 months	6.9 (5.2–11)	5.4 (4.4–13.8)	0.678	9
≥ 6 months	13.5 (7.6–43)	15 (7.15-60)	0.269	32

* Median, interquartile range; mg: milligrams; dL: deciliter; n: number of patients

The patients with type 1 diabetes and a follow-up of fewer than 6 months had a reduction in HbA1c levels (8.3 [6.9–10] vs. 7.7 [7-10.1], p = 0.363), while patients with a follow-up equal to or greater than 6 months had a smaller reduction in glycosylated hemoglobin levels (7.7 (7.12–8.5) vs. 7.515 [7–8.5], p = 0.345) (Table 4; Fig. 2). There were no differences in the other laboratory parameters of patients with type 1 diabetes (Table 4).

**Table 4:** Differences between the first and last metabolic parameter values, stratified by follow-up time in patients with type 1 diabetes

Type 1 Diabetes	First Teleconsultation	Last Teleconsultation	p-value	n
Glycosylated hemoglobin, %*				
< 6 months	8.3 (6.9–10)	7.7 (7-10.1)	0.363	15
≥ 6 months	7.7 (7.12–8.5)	7.5 (7-8.5)	0.345	42
LDL cholesterol, mg/dL*				
< 6 months	97 (74–120)	97 (87–107)	1	2
≥ 6 months	74 (58–89)	72 (61–77)	0.964	11
HDL cholesterol, mg/dL*				
< 6 months	46.5 (39–54)	56.5 (54–59)	0.180	2
≥ 6 months	66 (59–76)	63.5 (50–70)	0.240	10
Triglycerides, mg/dL*				
< 6 months	114 (108–120)	78 (60–96)	0.180	2
≥ 6 months	88 (57–106)	90 (65–135)	0.197	11
Creatinine, mg/dL*				
< 6 months	1 (0.64–1.08)	0.93 (0.73–1.13)	0.500	5
≥ 6 months	0.765 (0.675-0.9)	0.8 (0.735–0.875)	0.724	12
Microalbuminuria, mg/L*				
< 6 months	24.75 (17.5–32)	12 (12–12)	0.180	2
≥ 6 months	4.85 (2.55–14.5)	4.8 (4.4-14.05)	0.854	4

* Median, interquartile range; mg: milligrams; dL: deciliter; n: number of patients

Finally, evaluating the complications associated with diabetes mellitus that were identified through teleconsultation was proposed. Regarding patients with type 1 diabetes, 30.8% reported episodes of hypoglycemia, while patients with type 2 diabetes only 5.9% reported episodes of hypoglycemia. Patients admitted to the emergency room or receiving diabetes-related hospitalization accounted for less than 3% of the total number of patients. Only 1 person died as a result of diabetes (Table 5).

**Table 5:** Clinical outcomes or complications related to diabetes identified during the study period

Variable	Diabetes Mellitus	
	Type 1, n = 117	Type 2, n = 546
**Hypoglycemia (%)**		
Yes	36 (30,8)	32 (5,9)
No	78 (66,7)	370 (67,8)
ND	3 (2,6)	144 (26,4)
**Admission to the emergency room (%)**		
Yes	4 (3,4)	5 (0,9)
No	108 (92,3)	502 (91,9)
ND	5 (4,3)	39 (7,1)
**Need for hospitalization (%)**		
Yes	1 (0,9)	4 (0,7)
No	111 (94,9)	504 (92,3)
ND	5 (4,3)	38 (7)
**Mortality (%)**		
Yes	0	1 (0,2)
No	112 (95,7)	509 (93,2)
ND	5 (4,3)	36 (6,6)

ND: No data

## Discussion

### Principal findings

Patients with type 1 and type 2 diabetes who received follow-up by endocrinology teleconsultation maintained stable levels of glycosylated hemoglobin even if they had a follow-up of fewer to 6 months likewise, the other laboratory parameters that were used to evaluate cardiovascular risk and renal function, such as cholesterol, triglycerides, creatinine, and microalbuminuria, remained stable regardless of the follow-up time.

### Results in context

The World Health Organization defined telemedicine as “the delivery of health care services, where distance is a critical factor, by all health care professionals using information and communication technologies for the exchange of valid information for the diagnosis, treatment, and prevention of disease and injuries…” [20]. Chronic diseases, such as diabetes, require frequent medical visits to monitor and control the disease, as well as to receive therapeutic and lifestyle adjustments. However, telemedicine is a growing field that has been shown to improve self-management processes and clinical outcomes of care in patients with DM in a cost-effective manner [21].

Ruiz de Adana et al. conducted a randomized clinical trial with 330 patients with type 1 diabetes who were insulin users and were followed for six months. The intervention consisted of 3 appointments with an endocrinologist, conducted as telemedicine sessions or, for the control group in-person sessions. HbA1c remained stable compared with the initial value (7%) in both groups, with a percentage of variation in the telemedicine group of -0.4 (+/- 0.5%) and 0.01 ((+/- 0.6%) for the control group [22]. Our results support the efficacy of teleconsultation in the control of glycosylated hemoglobin in type 1 diabetes.

A randomized clinical trial by Rasmussen et al., with forty patients diagnosed with type 2 diabetes compared the results in the control of HbA1c, blood pressure, and lipid levels after six months of follow-up. The HbA1c and cholesterol levels were significantly lower in the telemedicine group than in the standard care group. The patients included in the study had a median HbA1c of 9.1% (7.6 mmol/mol) [23], while type 2 DM patients in our study had more controlled HbA1c levels, with an average of 6.6%, which could be because our patients experienced a minimal decrease or maintained stable values as evidenced in our results. Other studies even show that telemedicine interventions are more effective in reducing HbA1c in patients with type 2 DM, mainly when videoconferencing is used, with a frequency of intervention less than weekly and at least 6 months in duration [24, 25].

Sood et al. conducted another randomized clinical trial that included 288 subjects with type 1 and 2 diabetes to compare the effectiveness of endocrinologist’ care via video conference with that of face-to-face care. Again, patients had a -1.01% reduction in HbA1c in the telemedicine group and a -0.6% reduction in the face-to-face group (p = 0.19) and reported no differences in reducing cholesterol or serum creatinine levels. Another objective of this research was to evaluate user satisfaction, finding results similar to in-person care in terms of accessible communication, understanding by the specialist, and satisfaction with care. Clinical trials that compare the effectiveness of teleconsultation for the metabolic control of diabetic patients show that this modality of care is not inferior to in-person care, even with comparable results in patient satisfaction [26].

Telehealth intervention have enabled adequate metabolic control for patients with diabetes, as shown by optimal control of HbA1C, but many of the investigations that were performed in the follow-up of patients with diabetes used telemonitoring tools; this type of monitoring involves a technological infrastructure that collects and transmits clinical data remotely, allowing decisions to be made based on the values obtained [27]. A meta-analysis comparing telemedicine with the usual management concerning HbA1c levels and lipid profiles found an average difference of -0.31% (-0.37 to -0.24) in a median of 9 months (3–12 months) of follow-up in favor of telemedicine, with positive results in the reduction of LDL cholesterol. However, 13 studies used remote monitoring, and only three used video conferencing [28]. These monitoring instruments have enable reasonable glycemic control and reasonable control of other essential parameters patients with diabetes, especially those who use insulin, which can be maintained even after six months of follow-up, and they seem to enable better results when compared with the usual management techniques [29, 30].

In our study, we also describe clinical outcomes, and patients referred to hypoglycemic episodes mainly in the group of patients with type 1 diabetes. Clinical trials comparing telemedicine with in-person care did not report an increase in the number of cases of hypoglycemia reported by patients [22, 30], but telemedicine does not seem to have a protective effect against hypoglycemic episodes [27]. It is to be expected that patients with type 1 diabetes, who require treatment with insulin, will present with more episodes of hypoglycemia, given the drug’s mechanism of action and the strict glycemic monitoring [15], while patients with type 2 diabetes commonly use oral antidiabetic medications, and it is thus not necessary to perform blood glucose auto-monitoring with a glucometer for those patients. Nevertheless, a majority of patients reported ambulatory monitoring, and it is notably that this event was only recorded in 5.6% of patients with type 2 diabetes.

Only nine patients visited the emergency room, and five required hospitalization. A retrospective study compared patients with diabetes belonging to a telehealth program at home with diabetic patients who attended the same hospital network in person and who were followed for 4 years. They found that patients in the intervention group had fewer hospitalization events for preventable causes (0.7 vs. 1), a lower crude mortality rate (19.4% vs. 26.4%), and a longer survival time (1349 days vs. 1278 days). These results are possibly attributed to greater access to care needs by reducing patients’ geographical and transportation barriers [31]. To date, patients with noncommunicable diseases who use telehealth report a lower proportion of hospital admissions (telemedicine 42.9% vs. standard care 48.2%) when compared with the usual management (percentage difference − 10.8%, [95% CI -18.1% to -3.7%]) during 12 months of follow-up. This may indicate that telemedicine allows patients to manage their diseases better, and avoid exacerbations, while allowing specialists to identify early complications [32].

In our study, the percentage of patients who consulted the emergency department and required hospitalization was much lower than that reported in the literature for telehealth in general, so we consider that care via teleconsultation did not increase the need for secondary management. However, we highlight the necessity of investigations focused on adverse results and long-term outcomes of diabetes patients, who are followed exclusively via videoconference tools.

### Strengths and limitations

The retrospective nature of this study confers the limitation of the loss of some of the data. Additionally, 39.3% of patients with type 1 diabetes and 42.12% with type 2 diabetes received only one teleconsultation in the study period, which reduced the size of the sample avaible for evaluating changes in laboratory parameters. However, we identified adequate glycemic and metabolic control, regardless of the type of diabetes or the follow-up time. Because the study was conducted during mandatory lockdown, it lacked a control group and thus did not allow us to evaluate potential behavior differences between in-person modalities and our telemedicine context. This is the first study o this topic which involved patients from a Latin American population, which can serve as a basis for developing research focused on determining aspects that could affect the obtained results regarding barriers to access, satisfaction, and adherence from the perspective of patients.

## Conclusion

Our experience with teleconsultation allowed the follow-up of patients with type 1 and 2 diabetes to reach adequate metabolic control, independent of the follow-up time; additionally, it enabled the identification of exacerbations of the disease and avoided the need for secondary management. However, we consider it necessary to know users’ satisfaction with these new tools to reduce follow-up losses and incorporate this new modality into the routine care of chronic diseases. Considering the promising results reported in the literature thus far, it seems to be an option that will continue to be available in the future, and telehealth may become part of the integrated management for treating chronic noncommunicable diseases.

## Data Availability

The datasets analyzed during the current study and those that support the findings of this study are available from Fundación Valle del Lili, but restrictions apply to the availability of these data, due to internal privacy policies. Data are however available from Sangiovanni S. upon reasonable request and with permission of Fundación Valle del Lili.

## List of abbreviations

DM: Diabetes mellitus
HbA1c: Glycated hemoglobin
LDL: Low-density lipoprotein
HDL: High-density lipoprotein
